# The C3dg Fragment of Complement Is Superior to Conventional C3 as a Diagnostic Biomarker in Systemic Lupus Erythematosus

**DOI:** 10.3389/fimmu.2018.00581

**Published:** 2018-03-26

**Authors:** Anne Troldborg, Lisbeth Jensen, Bent Deleuran, Kristian Stengaard-Pedersen, Steffen Thiel, Jens Christian Jensenius

**Affiliations:** ^1^Department of Rheumatology, Aarhus University Hospital, Aarhus, Denmark; ^2^Institute of Clinical Medicine, Aarhus University, Aarhus, Denmark; ^3^Department of Biomedicine, Aarhus University, Aarhus, Denmark

**Keywords:** complement system, complement activation, systemic lupus erythematosus, biomarker, diagnostics

## Abstract

**Introduction/objectives:**

In 2012, hypocomplementemia was included in the classification criteria of systemic lupus erythematosus (SLE). The suggested measurement of C3 or C4 often reflect disease activity poorly. Our objective was to establish an assay measuring C3dg, which is generated following complement activation, and to evaluate the assay in a cross-sectional SLE cohort.

**Method:**

We included SLE patients (*n* = 169) and controls (*n* = 170) and developed a modified C3dg assay where C3dg fragments were separated from the large plasma proteins by polyethylene glycol (PEG), and the supernatant containing the C3dg fragment was used for analysis in an antibody-based sandwich-type assay. Gel permeation chromatography and western blotting were used to establish the optimal conditions for PEG precipitation.

**Results:**

16% PEG was optimal for separating C3dg from C3 and the larger protein fragments. The assay showed a high degree of stability when using EDTA plasma, and measurements correlated well with commercially available complement activation assays. SLE patients had higher concentrations in plasma of C3dg than controls (*p* < 0.05). ROC analysis showed that the C3dg activation fragment of C3 with an AUC of 0.96 (CI 0.94–0.98) was superior to C3 (AUC 0.52) in differentiating between patients and controls.

**Conclusion:**

Our results present a modified assay for the measurement of C3dg. We demonstrate that C3dg was superior to conventional C3 measurements in discriminating SLE patients from controls. We suggest that C3dg should be considered as a complement activation measurement in the SLE classification criteria.

## Introduction

Systemic lupus erythematosus (SLE) is an autoimmune disease involving loss of tolerance to self-antigens, which is manifested in the production of autoantibodies and deposition of complement-fixing immune complexes in injured tissue (1). Activation of the complement system has for decades been known as a significant contributor to SLE pathogenesis (2). However, not all mechanisms leading to complement activation are understood.

In 2012, new classification criteria of SLE were published by the SLICC group (Systemic Lupus International Collaborating Clinics) (3), and expert consensus included low complement protein 3 and 4 (C3 and C4) or low CH50 [complement hemolytic activity (4)] in the classification criteria. There is no explanation for this choice, but it reflects what has been done in the clinic for years. No suggestions of type of assays were given for the measurements of C3, C4, and CH50 in relation to the classification criteria.

The complement system, comprising more than 40 soluble and membrane bound proteins, is activated through three pathways: the classical, the alternative, and the lectin pathway (5). The classical and the lectin pathways are activated through pattern recognition. Serine proteases bound to a pattern-recognition molecule are activated upon recognition of a fitting pattern and through several enzymatic reactions this lead to the activation of the classical and lectin pathway C3-convertase (C4b2a) (6). The alternative pathway is in a state of constant activation, but is at the same time inhibited. There is a continuous hydrolyzes of C3 in the circulation, which potentially can lead to complement activation *via* the alternative pathway of complement activation. Under normal circumstances, this process is inhibited both in the circulation and at the cell surface (7, 8).

There are several ways to measure complement activation. One way is quantification of C3 and C4 protein (9) or fragments of the cleaved complement factors, e.g., C5a or C3a (10). Other assays estimate capacity of erythrocyte lysis (11) or estimate the level of soluble membrane attack complex (12, 13).

The central component in complement activation is C3 (14). When activated, C3 is cleaved into two fragments: C3a and C3b (14). C3b is further cleaved by factor I into iC3b and finally to C3dg and C3c (14). The smallest fragments C3a has a short half-live (15), while the larger fragment C3dg (37 kDa) has a longer plasma half-life of 4 h (16). C3c showed a shorter half-life than C3dg. Because of its size, C3dg can relatively easy be separated by size from the larger C3 molecules that also comprise the C3dg part [C3, C3(H_2_O), C3b, and iC3b]. These molecules will collectively here be termed C3′. A method for measuring the C3 split product C3dg was previously introduced using precipitation with 11% polyethylene glycol (PEG) to separate C3dg from C3′ (17). It has, however, not gained routine use. The same is the case for the modified rocket immuno-electrophoresis (18). The so-called double-decker rocket immuno-electrophoresis is used in a few clinical laboratories despite its technical challenges (19).

Our objective was to optimize an assay for the measurement of C3dg using precipitation with PEG followed by C3dg determination in the supernatant by immune assays. Furthermore, to evaluate if C3dg was superior to conventional C3 in discriminating between SLE patients and healthy controls in a cross-sectional cohort of SLE patients.

## Materials and Methods

### Patients

A cross-sectional cohort of 169 SLE patients were included consecutively at the out-patient clinic at the Department of Rheumatology, Aarhus University Hospital (November, 2015 to August, 2016). Inclusion criteria were fulfilment of the 1997 American College of Rheumatology (ACR) classification criteria for SLE (20), age 18 or above, understanding and speaking Danish. Exclusion criteria were infection, ongoing cancer treatment, and incapacitation. After written consent, clinical data including disease activity, SLE disease activity index (SLEDAI) (21), accumulated organ damage, SLICC/ACR (22), and treatment information were collected. Serum and plasma samples for research purposes were drawn at the same time as samples for routine biochemical assessments. Control samples (*n* = 170) were collected from blood donors at the blood bank of Aarhus University Hospital, Denmark (December, 2014 to January, 2015) as previously described (23).

Blood collected in EDTA plasma tubes (8 ml) and polystyrene serum tubes (10 ml) (Alere #367525 and #367896) were centrifuged at 2,000 *g* for 10 min. Plasma and serum were collected and frozen immediately at −80°C. Maximum time from blood drawing until freezing was 2 h.

### Assays for C3dg

#### Time-Resolved Immuno Fluorometric Assay (TRIFMA)

A standard for the assay was generated by activation of serum according to the recommendations of the study group for the manufacture of the International Complement Standard #2 (24). Ten milliliters of serum were incubated for 4 h at 37°C after the admixture of 1 ml heat aggregated human IgG (#007815; CSL Behring GmbH, Germany, 10 mg/ml TBS, aggregated at 63°C for 1 h) and 0.1 g zymosan (Sigma #Z4250). The activation was stopped by adding 550 µl 0.4 M EDTA and 200 µl Futhan (Sigma, 10 mg/ml H_2_O). Following precipitation with 11% PEG 6000 (w/v), the sample was centrifuged at 10,000 *g* 4°C for 30 min. The supernatant was collected and used as standard for the assay.

The standard curve was made by dilution of the activated serum standard 1/300 in Tris-buffered saline [0.14 M NaCl, 10 mM Tris, 14 mM sodium azide, with 0.05% (v/v) Tween 20 (TBS/Tween)], and further seven threefold dilutions.

Test samples (EDTA plasma) were pre-diluted 1/4 in TBS, and 40 µl was added to 60 µl TBS, 10 mM EDTA. Samples were kept on ice throughout mixing and dilution to inhibit complement activation. One hundred microliters of 32% PEG in H_2_O (w/v) were admixed and samples incubated for 1 h followed by centrifugation at 4,000 *g* 4°C for 15 min. The supernatants were withdrawn and diluted 1/200 in TBS/Tween, reaching a 2,000-fold dilution of the starting plasma sample. Duplicates of 100 µl were added to Fluoro Nunc MaxiSorb microtiter plates (Nunc, #437958 or #43791) previously coated by incubation overnight at room temperature (RT) with 100 µl rabbit anti-human C3dg (DAKO cat. no. A0063), mistakenly termed “anti-human C3d” (confirmed by correspondence with DAKO), at 5 µg/ml PBS and blocked by incubation with HSA at 1 mg HSA/ml TBS followed by wash with TBS-Tween. After each step, the wells were washed three times with TBS/Tween. Development after incubation overnight at 4°C was with the same anti-C3dg antibody as used for coating only now the antibody was biotinylated as previously described (25). One hundred microliters of biotin-anti-C3dg, 1 µg/ml TBS/Tween, were added to the wells and incubated 2 h at RT. After washing, 25 ng of Eu^3+^-streptavidin (Perkin Elmer #1244-360) in 100 µl TBS/Tween, 25 µM EDTA was added and incubated for 1 h at RT. After washing, 200 µl enhancement buffer (Ampliqon laboratory reagents #Q99800) was added to each well. Plates were read by time-resolved fluorometry using a DELFIA-reader Victor5 + (Perkin Elmer^®^) full TRIFMA protocol can be found Data Sheet 2 in Supplementary Material.

#### Enzyme-Linked Immune Sorbent Assay (ELISA)

The assay was also tested in an ELISA format identical to the above, except after incubation with biotinylated antibody and wash, 100 µl horseradish peroxidase (HRP)–streptavidin (DAKO #PO397) diluted 1/500 in TBS/Tween was added and incubated 1 h at RT. After washing, 100 µl substrate (Sigma #P4922-capsules and #A9941-tablets) was added and incubated for 30 min at 37°C. Plates were read at 405 nm on Victor5+ full ELISA protocol can be found Data Sheet 1 in Supplementary Material.

### Assay for C3′

The assay was performed analogous to the C3dg TRIFMA (excluding PEG precipitation). Wells were coated with rabbit anti-human C3c (DAKO #Q0368, 1 µg/ml TBS), and the development was with the same antibody biotinylated in-house at 1 µg/ml. The standard curve was constructed with dilutions of the serum pool assigned a value of 1 AU C3/ml. The test plasma samples were diluted 750,000-fold in TBS/Tw, 5 mM EDTA.

### Optimizing the PEG Precipitation

Plasma proteins were precipitated with increasing concentrations of PEG from 10 to 19% (w/v). Precipitations were done as in the assay described above with PEG being added at 20–38% (w/v). C3dg in the supernatants was estimated as described above.

### Gel Permeation Chromatography (GPC)

Samples of serum, supernatant of PEG precipitated activated serum and EDTA-plasma supernatant after precipitation with either 11 or 16% PEG (w/v), were subjected to GPC on a Superose 6 10/300 GL column (GE Healthcare). The running buffer was TBS/Tween, 5 mM EDTA. Samples were diluted 1:1 in buffer and 200 µl was loaded on the column. Fractions of 0.25 ml were collected in pure polystyrene microtiter plates (Nunc #249570), which were pre-blocked by incubation with TBS-Tween. C3dg in the fractions was quantified as described above.

### Western Blots

Samples of supernatants and precipitates after admixture of PEG were added to 1/4 volume sodium dodecyl sulfate-polyacrylamide gel electrophoresis (SDS-PAGE) sample buffer [30 mM Tris–HCl, 10% (v/v) glycerol, 8 M urea, 3% (w/v) SDS, 0.1% (w/v) bromophenol blue, pH 8.9]. TBS/Tween was added to reach the desired sample volume (30–45 µl). One-tenth volume of dithiothreitol (DDT), 0.6 M, was added to the samples to be reduced, and iodoacetic acid, 1.4 M, 1/10 vol, was added to all samples applied to gels containing both reduced and non-reduced samples. Samples were denatured at 100°C for 3 min. Proteins were separated on 4–15% gradient gels (Bio-Rad, Criterion TGX gels #567-1083). Following electrophoresis, the proteins were blotted onto nitrocellulose membranes (Bio-Rad #170-4159). The membranes were then blocked by incubation for 30 min at RT in TBS with 0.1% Tween (v/v), washed, and developed with polyclonal rabbit anti-human-C3dg (DAKO #0063) at 1 µg/ml or polyclonal rabbit anti-human-C3c (DAKO #0368) in primary buffer [Tris-buffered saline, 1 mM EDTA, pH 7.4, with 1 mg human serum albumin (CSL Behring #109697) and 100 µg human IgG (CSL Behring #007815) per ml]. Membranes were subsequently washed and incubated with HRP-conjugated goat anti-rabbit IgG antibody (DAKO #P0448) diluted 1/3,000 in secondary buffer (TBS/Tween, 100 µg human IgG/ml, 1 mM EDTA, pH 7.4). After washing, the blots were developed with SuperSignal West Dura extended-duration substrate (Pierce), and emission recorded by a charge-coupled device camera.

### Storage, Freeze/Thaw, and Diurnal Variation

To test the stability of the assay, regarding handling of samples, we initially tested both serum and EDTA plasma. Samples were tested after 0, 1 h, and 5 h at RT, and at 1 and 5 h at 37°C. Blood collected in EDTA was left for 5 h at RT or 5 h at 37°C before centrifugation and collection of plasma.

To test the stability of the samples to freezing/thawing cycles, a pool of EDTA plasma was aliquoted in 500 µl samples, frozen at −80°C, and thawed 1–9 times. Each thaw cycle was 1 h at RT.

Influence of diurnal variation on complement activation was investigated on six healthy individuals with samples taken at 4 h intervals through 24 h.

### Comparison With Commercial Assays

Immunoassays were bought from Hycult Biotech, estimating a C3c neo determinant (# HK368-02), Nordic Biosite, estimating a C3d neo determinant (#KSP-305) and Quidel, estimating C4d (#A008). Seventeen SLE samples and seventeen controls were randomly picked and run on all assays according to the manufacture’s description.

Twelve SLE patients were randomly picked to have C3d measured at the hospital of Vejle, Denmark, using a double-decker rocket immuno-electrophoresis method (19).

### Statistics

Checking the data for normality by Q–Q plots and histograms revealed that Gaussian distribution could not be assumed. Log-transformation did not improve normality significantly. Therefore, non-parametric tests were used for the statistical analysis. The Mann–Whitney *U*-test was used for comparison of plasma levels of the proteins in patients and controls and correlation analysis was performed calculating Spearman’s rank correlation coefficient. For comparison of repeated measurements, the Kruskal–Wallis test was used. *p*-Values <0.05 were considered statistically significant. Stata version 12 and GraphPad Prism software package (version 6.0) were used for data management and statistical calculations.

### Ethics

Clinical investigations were conducted according to the Declaration of Helsinki. The Danish Data Protection Agency and The Regional Committee on Health Research Ethics approved the study (case #1-10-72-214-13).

## Results

### Patients and Controls

The cohort of SLE patients (Table 1) was comparable to other Caucasian cohorts. Controls had a mean age of 45 (SD 15) at inclusion and 90% were female making them comparable to our SLE cohort with respect to age and gender.

**Table 1:** Systemic lupus erythematosus patient demographics.

Patient demographics	
Patient number	169
Age at inclusion, mean (SD)	45.4 (14.9)
Age at diagnosis, mean (SD)	33.5
Gender F (%)	90
Ethnicity: Caucasian (%)	97
**American College of Rheumatology (ACR) criteria (cumulative)**	
Number of ACR criteria, mean (SD)	6.3 (1.2)
Malar rash (ACR1) (%)	65.7
Discoid lupus (ACR2) (%)	5.3
Photosensitivity (ACR3) (%)	67.5
Oral/nasal ulcers (ACR4) (%)	44.4
Arthritis (ACR5) (%)	91.7
Serositis (ACR6) (%)	34.9
Nephritis (ACR7) (%)	31.4
CNS (ACR8) (%)	8.4
Hematological (ACR9) (%)	89.9
Immunological (ACR10) (%)	90.5
ANA (ACR11) (%)	98.2
**Clinical and biochemical data at time of inclusion**	
SLE disease activity index at inclusion, mean (SD)	3.9 (3.3)
SLICC, mean (SD)	0.9 (1.2)
**Treatment at time of inclusion**	
Hydroxychloroquine treatment (%)	79.6
Prednisolone treatment (%)	57.4
Other immuno-suppressives (%)	39.5

### Assay

**Figure 1:**
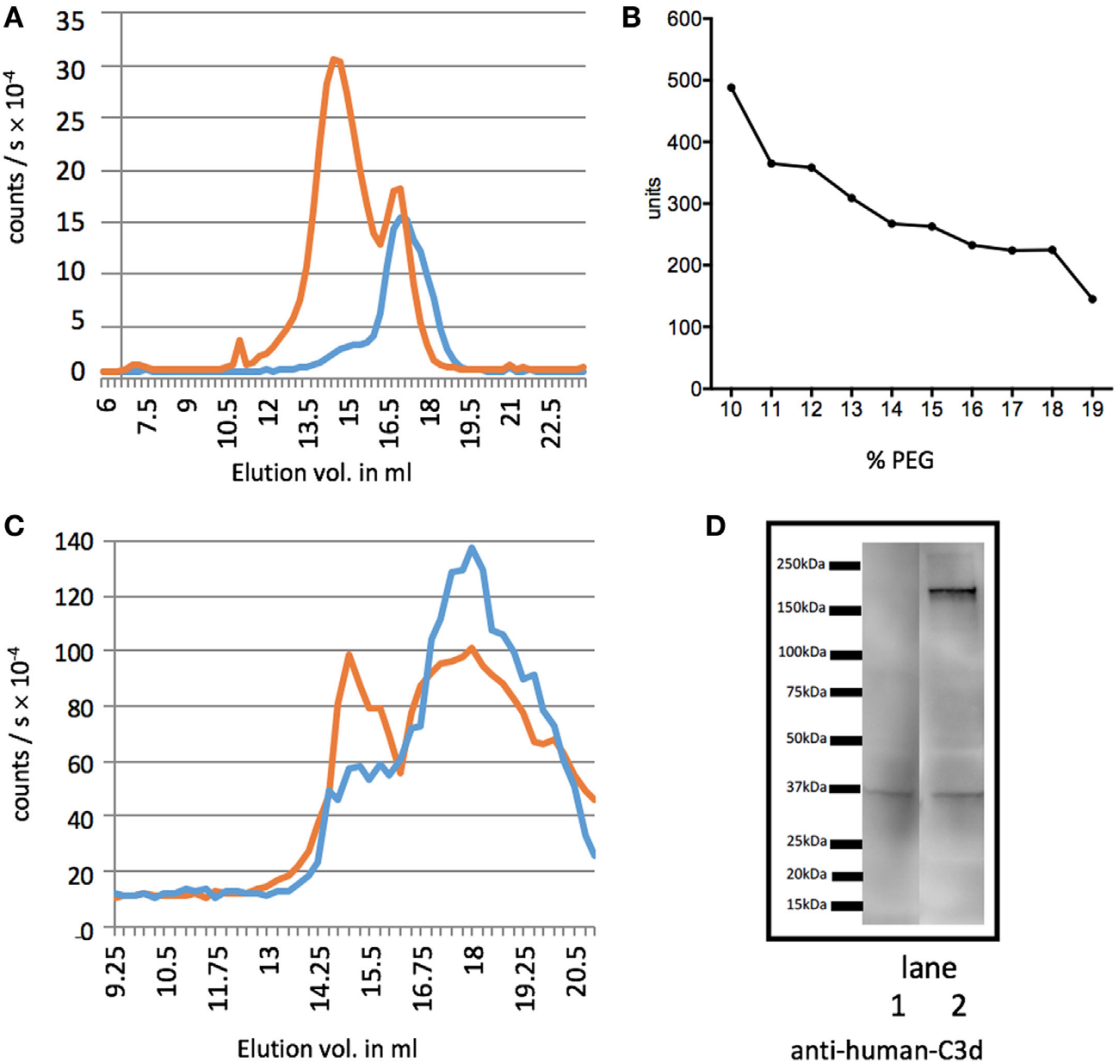
Separation of C3dg from the larger C3 fragments. Measurements of C3 molecules encompassing the determinants of the segment C3dg, i.e., C3 and all degradation molecules containing this part of C3. **(A)** The figure illustrates C3dg measurement on gel permeation chromatography (GPC) fractions of serum before activation (red line) and supernatant of polyethylene glycol (PEG)-precipitated activated serum (blue line). **(B)** Test for the optimal concentration of PEG for precipitation. Increasing concentrations of PEG were added to EDTA plasma and C3dg was estimated in the supernatants. **(C)** GPC of supernatant after precipitation of EDTA plasma with 11% (red) and 16% (blue) PEG. C3dg in the fractions was measured. The 11% supernatant shows two major peaks, the first corresponding to C3 and larger C3 components, and the second corresponding to free C3dg. After precipitation with 16% PEG the first was significantly reduced. Panel **(D)** shows the results of western blotting of supernatants of EDTA-plasma precipitated with 16% (lane 1) or 11% (lane 2) PEG. The samples (corresponding to 0.1 µl plasma) were run non-reduced on the SDS-PAGE. The blot was developed with anti-C3d antibody. It can be seen that the 11% PEG supernatant still contains appreciable amounts of larger C3dg-encompassing molecules. This was repeated three times with similar results.

To test whether the activation of our standard was successful, we ran GPC of non-activated serum and supernatant of PEG-precipitated serum (Figure 1A). Before activation (red) most C3dg is found in the large fragments (C3′), whereas after activation and precipitation of the activated serum, the supernatant (blue) contained only the smaller C3dg fragment. We further PEG-precipitated EDTA plasma to find out which PEG% would be the best to separate C3dg from C3′ (Figure 1B) and found a plateau at 16% PEG, suggesting this would be the optimal concentration.

To compare the previously suggested PEG concentration of 11% (w/v) to our choice of 16% (w/v), we ran GPCs of samples after precipitation with both PEG concentrations (Figure 1C) (17). Using 16% PEG-precipitation reduced the C3′ peak (blue Figure 1C) and showed the supernatant predominantly contained C3dg.

We then investigated the efficiency of precipitation with 11 and 16% PEG by western blotting. The result shown in Figure 1D illustrate that 16% PEG is superior to 11% in precipitating all fragments larger than C3dg. We observed that both free C3dg and larger C3dg-containing components, C3′, were present in the supernatant after PEG-precipitation with 11% (lane 2), whereas precipitation with 16% PEG (lane 1) yielded a much cleaner separation of free C3dg from the other components, supporting the results illustrated in Figures 1B,C. A more detailed analysis by western blot is presented in Figure S1 in Supplementary Material in which fragments of C3 is visualized in different samples developed with either anti-C3c or anti-C3d antibodies.

**Figure 2:**
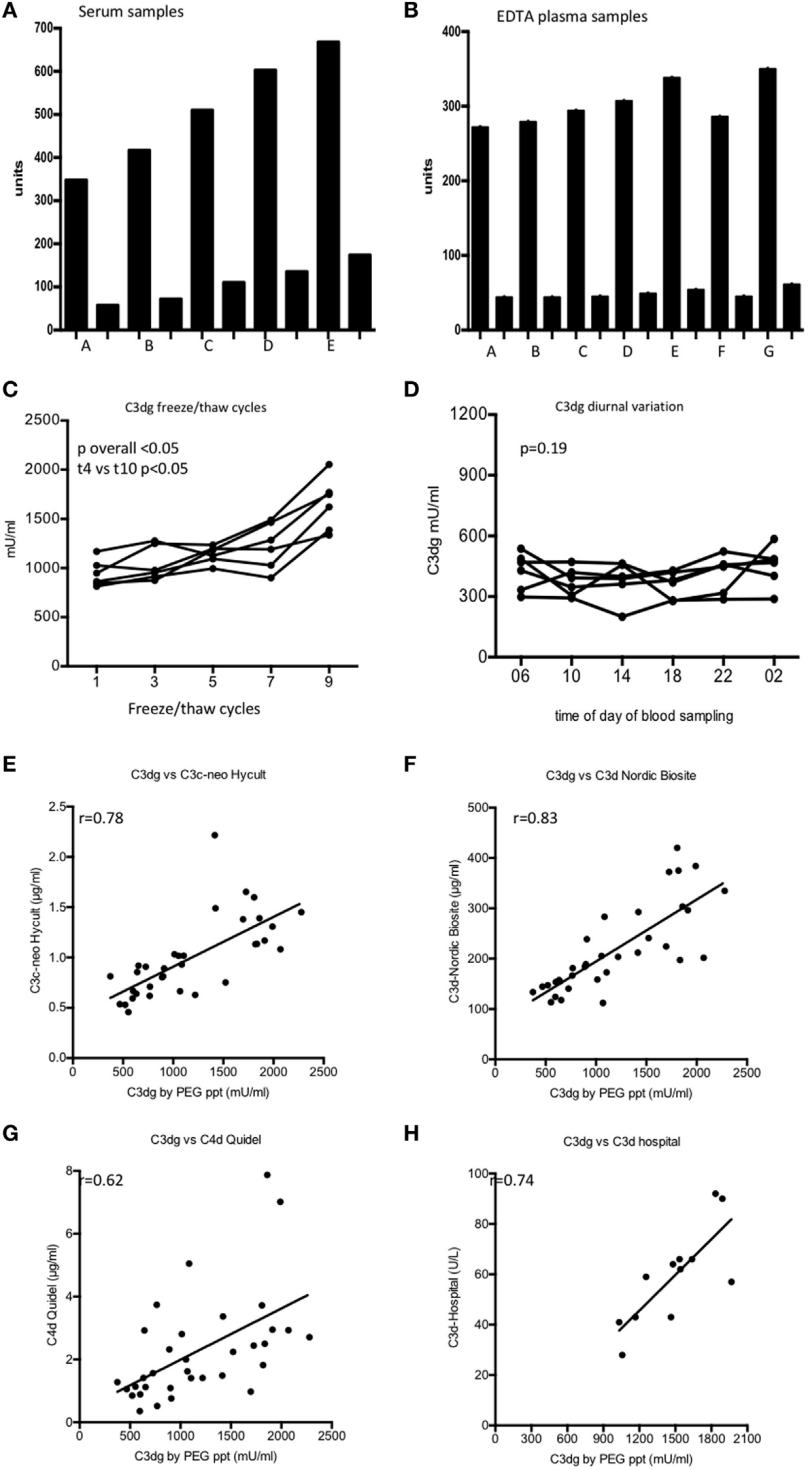
C3dg measured on samples handled in different ways from the time of blood withdrawal until measurement. **(A)** shows the result using serum and **(B)** shows the results using EDTA plasma. Each sample was diluted 100 (tall columns) and 1,000-fold (short columns). **(A)** Samples frozen *t* = 0, **(B)** samples frozen after 60 min at room temperature (RT), **(C)** samples frozen after 60 min at 37°C, **(D)** samples frozen *t* = 5 h at RT, **(E)** samples frozen *t* = 5 h 37°C, **(F)** EDTA plasma left 1 h at RT before centrifugation, **(G)** EDTA plasma left 5 h at RT before centrifugation. **(C)** The samples were tested for sensitivity to freeze/thaw cycles using six EDTA plasma samples. Samples were frozen up to 10 times each followed by thawing at RT for 1 h. C3dg was measured after 2nd, 4th, 6th, 8th, and 10th thawing. **(D)** C3dg diurnal variation. Six controls had blood drawn at six time points during 24 h, and C3dg was estimated. For comparison of the repeated measurements, the Kruskal–Wallis test was used. **(E)** Comparison between our assay and enzyme-linked immune sorbent assay (ELISA) kit from Hycult Biotech (C3c-neo determinants). **(F)** Comparison with ELISA kit from Nordic Biocite (C3d). **(G)** Comparison to ELISA kit from Quidel (C4d). 34 samples [17 systemic lupus erythematosus (SLE) and 17 controls] were analyzed in all kits. **(H)** Twelve randomly chosen SLE samples had C3d measured using the double rocket immuno-electrophoresis (19). The same samples were measured in our C3dg assay. To assess the correlation between the assays, Spearman correlation was used.

We observed that the concentration of C3dg in serum increased with both the time and the temperature to which the samples were exposed (Figure 2A). By contrast, no significant increase in C3dg was seen in EDTA plasma (Figure 2B). Freeze–thaw cycles of EDTA-plasma showed an increase in C3dg concentration in samples after four freeze–thaw cycles (*p* < 0.05) (Figure 2C). The concentration of C3dg in plasma did not display significant diurnal variation (*p* = 0.19) (Figure 2D).

The performance of our assay was compared to three commercially available assays for complement activation products (Figures 2E–G). Significant correlations (*p* < 0.05) were observed with all assays. Furthermore, our C3dg results were compared with the results obtained by double-decker rocket immuno-electrophoresis on plasma from 12 randomly picked patients (Figure 2H) (*r* = 0.74, *p* < 0.05).

**Figure 3:**
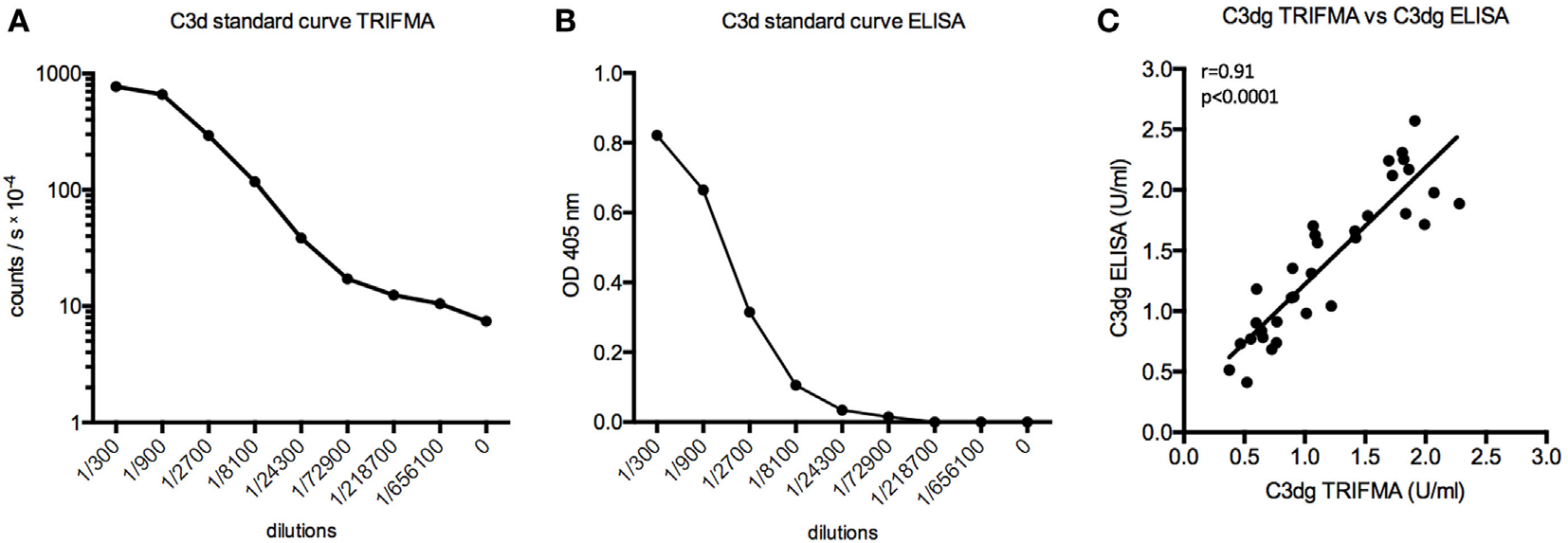
Time-resolved immuno fluorometric assay (TRIFMA) and enzyme-linked immune sorbent assay (ELISA) C3dg assays compared. **(A)** shows the standard curve of the assay carried out using europium-label (TRIFMA) and **(B)** as ELISA. **(C)** Correlation (Spearman) between measurements of C3dg on EDTA plasma samples using our assay as TRIFMA and as ELISA.

As ELISA is used more frequently than TRIFMA, it was considered expedient to subject the analysis of the PEG supernatants to assay by ELISA. The results were similar when comparing the two assays (*r* = 0.91, *p* < 0.0001) (Figure 3).

### Complement Activation in SLE Patients and Controls

**Figure 4:**
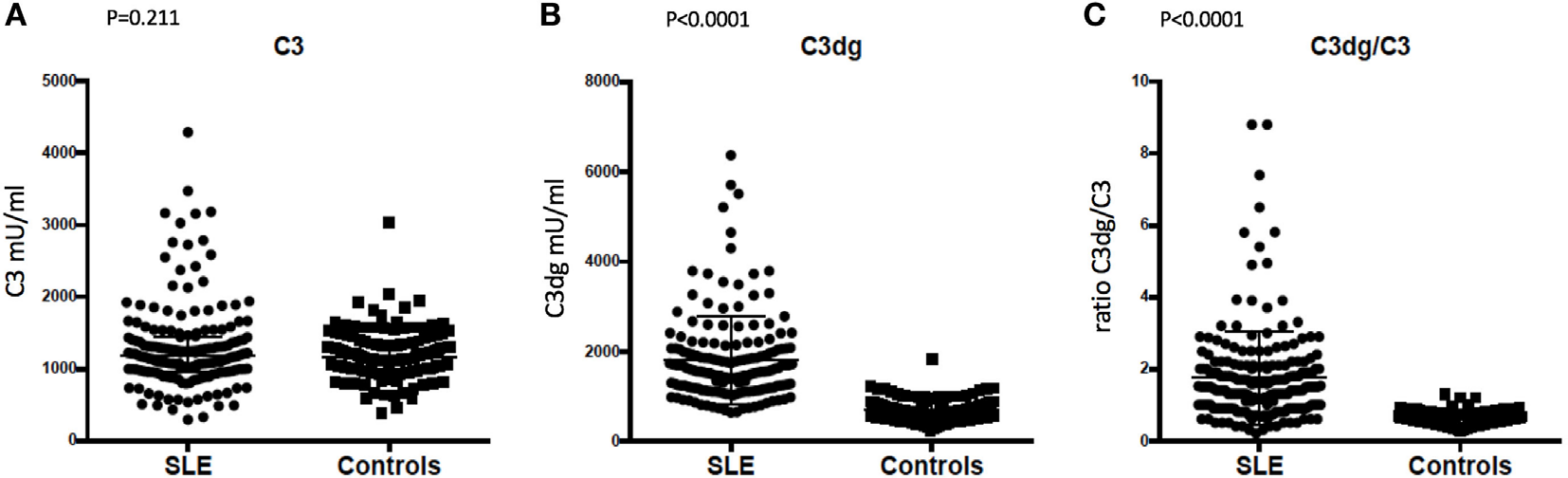
Comparison of concentrations in plasma of **(A)** C3, **(B)** C3dg, and **(C)** C3dg/C3 ratio in systemic lupus erythematosus (SLE) patients and healthy controls (measured by time-resolved immuno fluorometric assay). The Mann–Whitney *U* test was used for the comparison.

No significant difference was observed between SLE patients and controls for C3′, the value routinely determined as the C3 concentration (*p* = 0.211, Figure 4A). SLE patients showed higher C3dg concentrations in plasma compared with controls (*p* < 0.0001, Figure 4B). The C3dg/C3′ ratio was calculated for SLE patients and controls and showed a clear difference (*p* < 0.0001, Figure 4C).

C3dg and SLEDAI did not show any correlation (*r* = 0.03, *p* = 0.71). C3dg/C3′ correlated to SLEDAI (*r* = 0.28, *p* < 0.001) as did C3 (*r* = −0.49, *p* < 0.001), which was no surprise, since C3 is part of the SLEDAI score.

**Figure 5:**
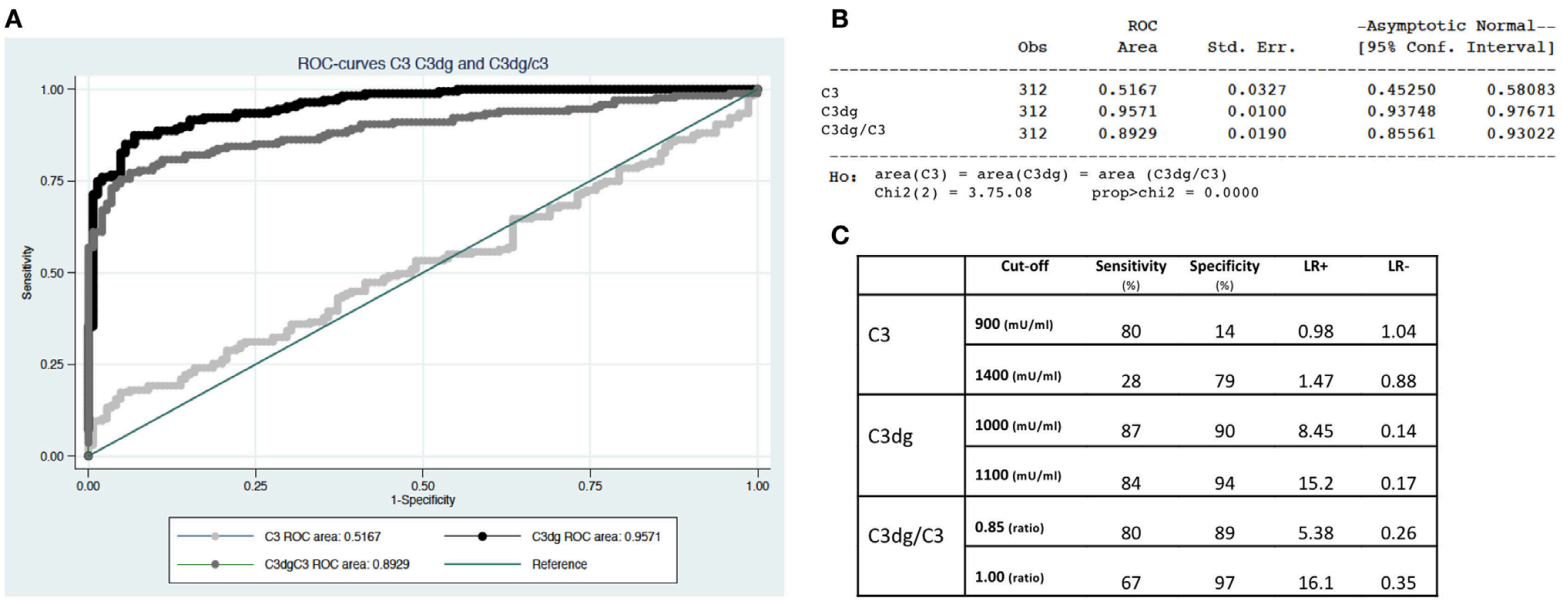
Separation of systemic lupus erythematosus patients from controls using C3, C3dg, and C3dg/C3. **(A)** demonstrates ROC-curves for C3 (light gray), C3dg (black), and C3dg/C3 (dark gray). **(B)** represents calculations of area under the curve with 95% confidence intervals for each of the curves in Figure 1B. **(C)** shows calculations of sensitivity, specificity, and likelihood ratios for C3, C3dg, and C3dg/C3 using two different cutoffs for each measurement.

ROC-curves were made based on the measurements of C3, C3dg, and C3dg/C3 (Figure 5). C3dg was superior in separating patients from controls with an area under the curve of 0.96 (CI 0.94–0.98) (Figures 5A,B). When estimating sensitivity and specificity based on different cutoffs, C3dg yielded the best combination of both high sensitivity and high specificity (Figure 5C), revealing a much larger complement turnover in SLE patients than reflected by the C3 measurements.

## Discussion

Low complement C3, C4, and CH50 were introduced into the classification criteria of SLE in 2012 (3), reflecting an international acceptance of the importance of complement in the diagnosis. However, in many cases of SLE, low C3 or C4 poorly reflect disease activity, as patients often present with low levels irrespectively of disease activity (26, 27). Our newly developed C3dg assay was clearly superior to the conventional C3 measurement in discriminating SLE patients from controls both with regards to specificity and sensitivity. Thus, C3dg may be a valuable diagnostic biomarker in SLE.

Instead of simply estimating C3 or C4 and interpreting low levels as evidence for complement activation or consumption, it seems more relevant to directly evaluate ongoing complement activation. Several assays have been published for estimating complement activation products without being widely implemented. With a half-life of 4 h, C3dg is an ideal candidate for evaluating ongoing complement activation. As mentioned in the Section “Introduction,” some procedures for estimating C3dg use precipitation of large proteins with PEG, followed by various assays for measurement of C3dg in the supernatant, have been published. The PEG concentration suggested in the first paper describing this idea (17), i.e., 11%, has been used in all the subsequent reports. We here re-addressed this issue and as illustrated with both GPC and western blot (Figures 1B–D), it appears expedient to increase the PEG concentrations to separate the large C3 components, here termed C3′, from C3dg (Figure 1). The PEG-C3dg assay has also been applied to samples from SLE patients. Significantly higher levels of C3dg have consistently been reported in patients compared with controls (11, 17, 19, 26, 28, 29). Also, the improvement gained using the C3dg/C3 ratio have been reported (11). Considering the sizable literature on this subject, it seems surprising that C3dg assays have received little clinical attention.

The C3dg estimation proved stable using EDTA-plasma. It is clear, however, that even with the addition of EDTA, complement activation is not completely inhibited unless samples are kept cold and not thawed multiple times. This underlines the necessity of handling samples similarly when comparing cohorts (30). Control samples were stored for approximately a year longer than patient samples, and at least for EDTA plasma, storage at minus 80°C seemed to stop complement activation, as controls displayed considerable lower C3dg than patients. Another concern, when using blood for measurements is whether or not the component of interest displays diurnal variation. This is the case for several proteins and hormones (31, 32). We, however, observed no significant diurnal variation in complement activation reflected by C3dg.

The plasma concentration of C3 and C4 show inter-individual variation (33). Concentrations depend to a large extend on synthesis; however, SLE patients often show low levels of C4 due to a partial genetic defect (34, 35). Low C4, therefore, does not necessarily reflect consumption. Thus, it is dubious to use a single complement measurement for diagnostic/clinical purposes. Intuitively, therefore, it makes sense to use the C3dg/C3′ ratio, as previously suggested by Röther et al. (29). If initial concentration of intact complement components influences the concentration upon activation, it is reasonable to correct for this by calculating the C3dg/C3 ratio. Whether this measurement can be used for clinical purposes, e.g., to rule out a flare, remains to be tested on a prospective cohort. We demonstrated that the use of C3, as suggested in the SLE classification criteria, was not optimal for the SLE diagnosis. We found that C3dg was significantly higher in SLE patients than in healthy controls, whereas this was not the case for C3. C3dg showed superiority with regard to both sensitivity and specificity compared with C3. The performance of C3dg as a criterion for classification of SLE should be further evaluated by comparing SLE with other diseases.

The study raises the question of why most SLE patients have higher C3dg concentrations in plasma than controls (Figure 4). It likely indicates that in most patients there is complement turnover, even under conditions where patients are regarded as having inactive disease (36). The alternative pathway is an essential amplification loop in the activation of the complement system (37, 38). We know that lack of control of the alternative pathway potentially leads to devastating disease (39, 40). A possible explanation for higher C3dg in patients could be a relative lack of inhibition of the alternative pathway, making it easier to tip the balance to non-inhibition in cases of infection, cancer, sun exposure, and trauma, all known causes of SLE flares. Studies on lupus-prone mice have demonstrated exacerbation of disease with inadequate cell level complement inhibition (41). In line with this, low factor H has been associated with lupus nephritis (42). Another possible explanation could be anti-C3-antibodies. As reported by Vasilev et al., anti-C3-antibodies bind to the C3c part of C3 and thus bind C3, iC3b, C3b, and C3c (43). The antibodies likely compete for the same binding site as factor H, thereby hindering inhibition at the cell surface (43). Thus, one would expect patients with anti-C3-antibodies to have higher levels of C3dg because of more complement activation. Future studies are warranted to elucidate this in SLE patients.

We present a modified assay for measuring C3dg. The assay is simple, inexpensive, and stable. The estimation of C3dg directly reflects complement turnover independently of activation pathway. The assay differentiated excellently between SLE patients and healthy controls. We propose that C3dg measurements should be evaluated as a standard complement activation measurement in patients with SLE particularly with respect to classification.

## Ethics Statement

Clinical investigations were conducted according to the Declaration of Helsinki. The Danish Data Protection Agency and The Regional Committee on Health Research Ethics approved the study (case #1-10-72-214-13).

## Author Contributions

AT and LJ performed the laboratory experiments. AT was in charge of collecting blood samples and handling the blood samples after they were drawn. AT, BD, and KS-P handled patient inclusion and clinical assessments. ST and JJ developed the assays used in the project and supervised laboratory procedures. AT, ST, JJ, and KS-P wrote the manuscript. All authors participated in the editing of the article.

## Conflict of Interest Statement

The authors declare that the research was conducted in the absence of any commercial or financial relationships that could be construed as a potential conflict of interest.
