# Supplementary material for: The C3dg Fragment of Complement Is Superior to Conventional C3 as a Diagnostic Biomarker in Systemic Lupus Erythematosus
Source: Front Immunol. 2018 Mar 26;9:581. doi: 10.3389/fimmu.2018.00581 (PMC5879092; doi:10.3389/fimmu.2018.00581)
Supplement: Supplementary file 2 [file data_sheet_2.docx]

Supplementary Material

- **The C3dg fragment of complement is superior to conventional C3 as a diagnostic biomarker in Systemic Lupus Erythematosus**
- Anne Troldborg, Lisbeth Jensen, Bent Deleuran, Kristian Stengaard-Pedersen, Steffen Thiel, Jens Christian Jensenius

Corresponding Author: annetrol@rm.dk

# Supplementary Data

## TRIFMA (Time Resolved Immuno Fluoro Metric Assay) for Complement Component C3dg

Assay for ongoing complement activation in vivo: The assay is based on the removal of large C3 components from plasma by precipitation with PEG, followed by quantification of the small C3dg component in the supernatant by sandwich immunoassay

## Materials

1. FlouroNunc MaxiSorp microtitre plates, # 446612, Nunc, Roskilde, Denmark.
2. Polyclonal rabbit anti-human C3d, # A0063, DAKO, Copenhagen, Denmark. This is actually anti-C3dg as it is raised against the naturally processed fragment purified from serum (personal communication with DAKO). It is the IgG fraction of anti-C3dg antiserum.
3. Detergent-blocked reservoir: The reservoir for multi-pipetting is filled with Tween 20 at 0.1% in H_2_O and incubated at RT for 30 min, washed thoroughly with H_2_O, and air dried.
4. Anti-C3d coated micro-titre plates: anti-C3d is diluted to 5 µg/ml PBS in detergent-blocked reservoir. 100 µl is added to each well and incubated overnight at RT.
5. The wells are emptied, filled with 200 µl of 1mg HSA/ml TBS and incubated for 1 hour at RT.
6. The wells are washed with TBS/Tween 3 times. The plates may then be kept for up to four months at 4°C with the wells filled with 200µl TBS.
7. 1.5 ml Eppendorf micro tubes, e. g., EasyCap, Sarstedt, cat# 72.690.550.
8. 32% (w/v) PEG 6000, Merck #8.07491.1000 in H_2_O.
9. TBS: Tris-buffered saline, 0.14 M NaCl, 10 mM Tris, 0.09% (w/v) sodium azide, pH 7.4.
10. 1 mg HSA/ml TBS. We use **h**uman **s**erum **a**lbumin, 200 mg/ml, Statens Serum Institut, Copenhagen
11. TBS/Tween: TBS with 0.05% (v/v) Tween-20 (Merck 8.17072.1000).
12. TBS/EDTA: TBS, 10 mM EDTA.
13. Heat aggregated human IgG: #007815; CSL Behring GmbH, Germany. 10 mg/ml TBS, aggregated at 63°C for 1h.
14. Plasma samples from venous blood collected in EDTA. Samples are centrifuged at 2000*g* for 10 minutes at room temperature within 1-2 hours of withdrawal, aliquoted, and stored at -80 °C
15. Standard:

The standard for the assay is generated by activation of serum according to the recommendations of the study group for the manufacture of the International Complement Standard #2 (ICS#2). To ten ml of serum is added 1 ml heat aggregated human IgG and 0.1 g zymosan (Sigma #Z4250). This is incubated for 4h at 37°C. The activation is stopped by adding 550 µl 0.4 M EDTA and 200 µl Futhan (Sigma, #N0289, 10 mg/ml H_2_O). An equal vol. of 22% (w/v) PEG 6,000 in H2O is added under stirring. After 30 min on ice the mixture is centrifuged at 10,000*g*, 4°C, for 30 minutes. The supernatant is collected and used as standard for the assay. Samples of 20 µl are kept at -70°C or -80°C.

1. Quality controls: three internal controls (K1, K2, K3) are included on each assay plate to control for inter-assay variability. The controls are made from 20 ml of serum, which is activated with heat aggregated IgG and zymosan as under point 15.

In order to cover the range of C3dg encountered in plasma samples control 1 is activated serum diluted 1/30 in TBS/EDTA, control 2 is diluted 1/160, and control 3 is diluted 1/1000.

The controls are aliquoted to Eppendorf tubes at 100 µL/tube and are kept at -70°C and are ready to be use in the assay by the addition of 100 µl PEG 32% in parallel with the test plasma samples.

1. Biotin-labelled rabbit anti-human-C3d: The antibody from DAKO is biotinylated with biotin-N-hydroxysuccinimide (BNHS, Sigma # H1759). Before the addition of BNHS, the antibody is dialysed 2 times in PBS pH 7.5 and once in PBS adjusted to pH 8.5 with 5% (w/v) sodium carbonate. 167 µL of a 1 mg BNHS/ml DMSO and 1 mg antibody in 1 mL PBS, pH 8.5, are mixed and incubated for four hours at room temperature followed by dialysis against TBS. Storage at 4°C.
2. TBS/Tween/EDTA: TBS/Tween with 25 µM EDTA
3. Europium-labelled streptavidin (Perkim Elmer, State, USA, # 1244-360, 0.25mg/2.5 ml).
4. Enhancement buffer (Ampliqon laboratory reagents, State, USA? # Q99800)

**Procedure**

All reagents and samples are used at 100 µl per well.

1. Mix the thawed vials with the standard pool, the quality controls and the test samples thoroughly on a whirly-mixer.
2. PEG precipitation of EDTA-plasma samples:

Prepare trays with crushed ice and place here samples, buffer and PEG in order to carry out the precipitation in the cold (to inhibit enzyme reactions).
Add 10 µl of sample to 90 µl TBS, 10 mM EDTA. Mix. Dilute 1/100: 10 µL to 990 µL buffer. Mix.
Add 100 µl of 32% PEG (i.e., 16 % PEG precipitation)
Mix thoroughly.
Leave on ice for 1 hour.
Centrifuge samples for 15 minutes at 4000*g* at 4°C.

The quality controls are handled a bit differently as they are already prepared for precipitation and frozen in 100 µl volumes. They are thawed, mixed, and 100 μl 32% PEG is added. Mix thoroughly and leave on ice for 1 hour. Centrifuge samples for 15 minutes at 4000*g* at 4°C.

10 µl supernatant of samples and controls are withdrawn and diluted in 990 µl TBS/Tween (1/100), reaching a final sample dilution of 2000-fold of the starting plasma sample.

1. To produce the standard curve the standard plasma is diluted 1/300 in TBS-tween and further seven three-fold dilutions (ex. - st. # 1: 1/3 (15 µl standard plasma + 30 µl buffer), st. # 2: 1/300 (10 µl st 1 + 990 µl buffer), st. # 3:1/900 (200 µl st2+ 400 µl buffer) etc.).
2. All samples (+ standard and controls) are to be added in duplicates to the anti-C3dg coated wells. We add the standards in the first 16 wells, TBS/Tween in the next 2 wells and the controls in the following 6 wells. Finally, the test samples are added. Incubate the plate overnight at 4°C.
3. Wash three times with TBS/Tween.
4. Add 100 µl freshly diluted Bio-anti-C3dg, 1 μg/ml in TBS/Tween.
   Incubate for 2 hours at RT.
5. Wash three times with TBS/Tween.
6. Add 100 µl europium- streptavidin freshly diluted 1/1000 in TBS/Tween/ 25 µM EDTA. Incubate for 1 hour at RT.
7. Wash three times with TBS/Tween and then add 200 µl Enhancement solution.
8. Shake for 5 minutes on a plate shaker.
9. Read the counts by time-resolved fluorometry (*e.g.,* using the Victor5 DELFIA-reader from Perkin Elmer).
10. The plate may be read at any time convenient.

**
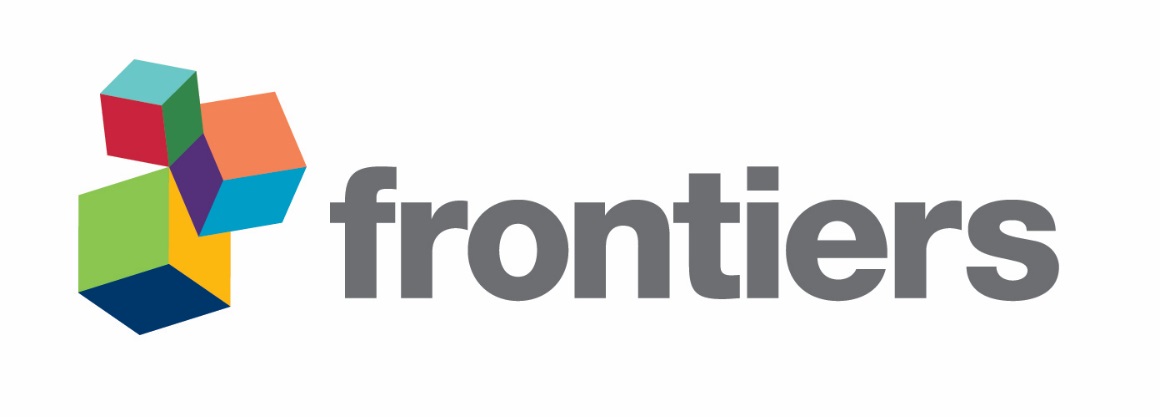
**

.
