# Supplementary material for: The C3dg Fragment of Complement Is Superior to Conventional C3 as a Diagnostic Biomarker in Systemic Lupus Erythematosus
Source: Front Immunol. 2018 Mar 26;9:581. doi: 10.3389/fimmu.2018.00581 (PMC5879092; doi:10.3389/fimmu.2018.00581)
Supplement: Supplementary file 3 [file presentation_1.PDF]

Supplementary Material

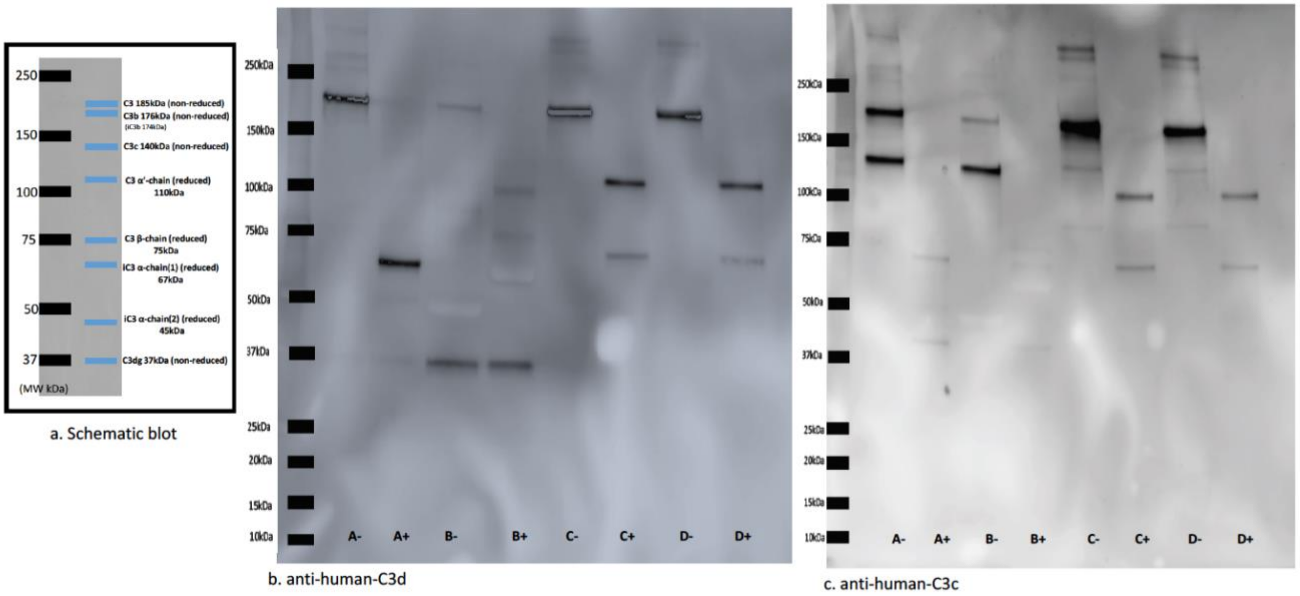

Supplementary Figure 1. Western blots illustrating C3 and its fragments containing either C3c or C3dg in different samples. Blots were developed using rabbit anti-human-C3d (DAKO) and rabbit anti-human-C3c (DAKO). Samples on blots: A. Activated serum (0.01 µl), B. Supernatant of activated serum after precipitation with 11% PEG (0.1 µl), C. EDTA plasma (0.01 µl), D. Re-suspended pellet from EDTA plasma after precipitation with 16% PEG (0.01 µl), E) Supernatant of EDTA-plasma(SLE) after precipitation with 11% PEG (1.5 µl) and E. Supernatant of EDTA-plasma (SLE) after precipitation with 16% PEG (2.4 µl). (+ denotes reduced sample, - denotes non-reduced sample). In **a,** a schematic overview of where to identify different C3 fragments on a blot is given. Blot **b** and **c** illustrates which C3 fragment the antibodies used in the article (anti-C3dg and anti-C3c) identifies. Exposure time for b. and c. was 20 sec.
